# The Use of End-Tidal CO_2_ and Integrated Pulmonary Index to Predict Postspinal Hypotension in Cesarean Section

**DOI:** 10.3390/jcm13010085

**Published:** 2023-12-23

**Authors:** Emine Aslanlar, Camille Kamel Alharach, İnci Kara, Ozkan Onal, Durmuş Ali Aslanlar

**Affiliations:** 1 Department of Anesthesiology, Medicine Faculty, Selcuk University, Ardıçlı, Akademi, Celal Bayar St. No. 313, Konya 42250, Turkey; drincikara@yahoo.com (İ.K.); drozkanonal@gmail.com (O.O.); 2 Department of Anesthesiology, Medicine Faculty, Başkent University, Hocacihan Mahallesi Saray Caddesi No. 1, Konya 42080, Turkey; calharach@hotmail.com; 3 Meram State Hospital, Hacışaban, Yeni Meram St. No. 97, Konya 42090, Turkey; daaslanlar@yandex.com

**Keywords:** cesarean section, end-tidal CO_2_, integrated pulmonary index, postspinal hypotension

## Abstract

Early diagnosis and treatment of postspinal hypotension (PSH) in obstetric anaesthesia reduces the risk of maternofetal complications. In this study, the effect of EtCO_2_ and the integrated pulmonary index (IPI) in predicting PSH was investigated. Patients scheduled for cesarean section under spinal anaesthesia were included. The Capnostream 35 respiratory monitor (Medtronic, Inc., Dublin, Ireland) was used for EtCO_2_ and IPI. PSH developed in 52 (63.4%) of the 82 patients. EtCO_2_ and IPI values decreased significantly compared with baseline values in patients who developed PSH. There were statistically significant differences in EtCO_2_ (*p* = 0.001) and the IPI change (*p* = 0.045) in patients who developed PSH compared with those who did not. It was found that the EtCO_2_ difference had an independent effect on predicting PSH (*p* < 0.05), whereas the IPI difference did not (*p* > 0.05). One unit decrease in EtCO_2_ from the baseline increased the risk of PSH by 3.3 times. ROC curve analysis showed that the magnitude of change in EtCO_2_ was diagnostic for predicting PSH (AUC: 0.90 (0.83–0.97; *p* < 0.001)). IPI showed no predictive value for postspinal hypotension in cesarean section. However, EtCO_2_ monitoring, which is non-invasive and real-time monitoring, can be used to predict postspinal hypotension.

## 1. Introduction

Spinal anaesthesia is frequently preferred in caesarean sections because it provides safe and rapid anaesthesia. The most important and frequent complication of spinal anaesthesia is hypotension due to sympathetic blockade. The incidence of postspinal hypotension in non-pregnants is 15.3–33%, whereas in pregnant women it is much higher, reaching up to 70% [1]. Aortocaval compression due to the gravid uterus, increased sensitivity to local anaesthetics, decreased basal vasomotor tone due to using substances with strong vasodilator properties such as progesterone, relaxin and nitric oxide, which increase during pregnancy, and sympathetic blockade induced by spinal anaesthesia are possible underlying factors for the sudden onset and severe hypotension observed in parturient women receiving spinal anaesthesia. Untreated PSH may cause ischaemic events and organ hypoperfusion.

PSH may cause the loss of consciousness and aspiration in the mother and may also cause fetal hypoxia, fetal acidosis and low Apgar scores due to decreased uteroplacental blood flow. As a result, it is crucial for anaesthetists to identify PSH early and treat it effectively during cesarean sections to protect both the mother and fetus from these serious complications [2].

The integrated pulmonary index is an algorithm used to assess the patient’s respiratory status. This algorithm uses real-time measurements and the interactions of four parameters, EtCO_2_, RR, HR and SpO_2_. The IPI measurement is based on the continuous conversion of these four parameters into a single index from 1 to 10, where “10” indicates a normal respiratory state and “1” indicates the need for immediate action on the part of the patient. On the monitor, this index value can be shown continually as waveform or numerical data. The following criteria were used to assess how the values and patient status related: 10 = Normal, 8–9 = Within normal range, 7 = Close to normal range; needs attention, 5–6 = Needs attention and may require intervention, 3–4 = Needs intervention, and 1–2 = Needs immediate intervention [3]. IPI monitoring has been used mostly for monitoring ventilation in procedures under sedoanalgesia [4,5,6]. Because it provides for a non-invasive, dynamic and in-the-moment measurement, represents the respiratory condition with high specificity and sensitivity and can help identify respiratory issues early on, this monitoring has drawn interest [3,6].

EtCO_2_, one of the four parameters considered by the integrated lung index value, is a cardiac-output-related parameter that also assesses the efficiency of ventilation. CO_2_ delivery to the lungs also depends on cardiac output. Thus, studies have demonstrated a decrease in EtCO_2_ due to reduced cardiac output, as observed under conditions of hypotension and hypovolemia [7,8]. The decrease in preload and afterload due to sympathetic blockade caused by spinal anaesthesia leads to a decrease in cardiac output and PSH [9]. The decrease in EtCO_2_ with the decrease in cardiac output may cause a change in IPI. Therefore, we thought that this change in IPI may be a new parameter that can be used to predict PSH. Therefore, we aimed to demonstrate the efficacy of a non-invasive method that can detect PSH early, provide instantaneous values and be non-invasive by monitoring EtCO_2_ and IPI during the caesarean section.

## 2. Material Methods

Ethical approval for this study was granted by the Ethics Committee of University Selçuk Medicine Faculty (date: 12 October 2021/No: 2021/446). It has been registered with ClinicalTrials.gov (NCT05237856). The patient was included from February 2022 to November 2022. Informed consent was obtained from all individual participants included in the study. This study was performed in line with the principles of the Declaration of Helsinki.

Eighty-two patients aged 18 years and older with ASA II physical status scheduled for elective cesarean section under spinal anaesthesia were included in the study. Pregnant women who refused to participate in the study underwent emergency caesarean section or general anaesthesia and had heart disease, pulmonary embolism or chronic respiratory disorders, and ASA > II were excluded. In addition, cases that met the study conditions but failed spinal anaesthesia or returned to general anaesthesia due to inadequate block were excluded from the study.

Demographic data, including age, height, weight, body mass index, comorbidities, gestational hypertension, and gestational diabetes mellitus, were collected. Patients were placed in a 15° left lateral tilt position to prevent aortic-caval syndrome. Standard monitoring was carried out, including non-invasive blood pressure readings, pulse oximetry, and electrocardiography. Systolic blood pressure (SBP), diastolic blood pressure (DBP) and mean arterial pressure (MAP) were measured and recorded every two minutes until the baby was born. Two peripheral intravenous lines were opened, and isotonic solution was co-loaded (10 mL/h).

Capnostream 35 Portable Respiratory Monitor (Medtronic, Inc., Dublin, Ireland) was connected to the patient for assessment of IPI and its components EtCO_2_, RR, SpO_2_ and HR. This monitor collects SpO_2_ and HR measurements via pulse oximetry using a finger probe. Additionally, it monitors EtCO_2_ and RR via a nasal cannula that allows for oral and nasal sampling of inspired and exhaled air. This monitor utilises microstream capnography with proprietary molecular correlation spectroscopy technology using a CO_2_-specific infrared wavelength when measuring EtCO_2_. The capnography sampling line consists of a nasal cannula inserted just inside the nostril and an oral scoop attached to this nasal cannula. Oral scoop design has wide surface area to provide breath capture even in the presence of shallow breathing. This special design, consisting of a nasal cannula and an oral scoop, allows the sampling line to take CO_2_ samples from both the mouth and the nose. The IPI, EtCO_2_, RR, SpO_2_ and HR were measured and recorded continuously with the monitor and the values before spinal anaesthesia were accepted as baseline values. In the sitting position, 9–10 mg of 0.5% hyperbaric bupivacaine (0.06 mg/cm height) [10] and 10 mcg of fentanyl were administered intrathecally by entering the subarachnoid space through the L3–L4 or L4–L5 interval with a 25-gauge Quincke tipped needle, following antiseptic rules. After spinal anaesthesia, the patient was placed in the supine position. The level of sensory block was evaluated with a cold swab and surgery was initiated when the level of sensory block reached the T6 dermatome or higher.

Non-invasive blood pressure readings taken before spinal anaesthesia served as the baseline, and PSH was defined as a decrease in blood pressure of 20% or more or a drop in systolic blood pressure below 100 mmHg. PSH was treated with intravenous ephedrine. Rescue ephedrine 10 mg was administered as a bolus in each hypotension episode. A heart rate < 55 was considered bradycardic, and atropine 0.5 mg was administered.

The aim of our study was to investigate the correlation between PSH and EtCO_2_ and IPI measurements and to determine the rate of PSH.

### Statistical Analysis

The research data were computerised and analysed using SPSS for Windows 22.0. Variables were presented as mean ± standard deviation (minimum–maximum), frequency distribution and percentages. The Pearson chi-square test and Fisher’s exact test were used to evaluate categorical variables. Visual (histogram and probability plots) and analytical (Kolmogorov--Smirnov test/Shapiro--Wilk test) methods were used to assess the conformity of variables to the normal distribution. Mann--Whitney U test, Wilcoxon signed rank test, Student’s *t*-test and paired sample *t*-test were used as statistical methods according to the conformity to normal distribution. In multivariate analysis, logistic regression analysis was used to investigate independent predictors of postoperative hypotension using the possible factors identified in the previous analyses. The discriminatory power of the EtCO_2_ difference in predicting postspinal hypotension was assessed via receiver operating characteristic (ROC) curve analysis.

## 3. Results

A total of 82 patients undergoing a caesarean section under spinal anaesthesia were studied. PSH developed in 63.4% of patients. There was a statistically significant difference in age between those who developed PSH and those who did not (*p* = 0.025). Female patients who developed PSH were significantly younger than those who did not. There was no statistically significant difference in demographics other than the age gap between patients who developed PSH and those who did not (*p* > 0.05) (Table 1).

While a statistically significant difference was found between patients with and without PSH in terms of baseline SpO_2_ values (*p* < 0.05), no significant difference was found between the two groups in terms of baseline EtCO_2_, RR, HR and IPI values (*p* > 0.05) (Table 1).

The change between the baseline IPI and its component parameters of the patients who developed PSH and the parameters measured at the time of PSH development is shown in Table 2. While EtCO_2_ and IPI values measured at the time of development of hypotension in patients who developed PSH decreased significantly compared to baseline values, SpO_2_ value increased significantly (*p* < 0.05); RR and HR values did not show a significant change compared to baseline values (*p* > 0.05) (Table 2).

The mean time from spinal anaesthesia to the development of PSH was 3.6 ± 1.8 (min–max: 1–8) minutes. The mean time from the development of postspinal hypotension to reaching a normotensive state was 3.2 ± 1.4 (min–max: 1–7) minutes. Of the 52 patients who developed PSH, 12 patients had one episode of hypotension, 26 patients had two episodes, 10 patients had three episodes and 4 patients had four episodes of hypotension. The mean number of episodes was 2.11 ± 0.85.

As the median time from the application of spinal anaesthesia to the development of PSH in the study was found to be 3 min, the measurements of the patients who developed PSH and those who did not at 3 min were compared. While there was no statistically significant difference between the two groups for RR, SpO_2_ and HR change (*p* > 0.05), there were statistically significant differences for SBP, DBP, MAP, EtCO_2_ (*p* = 0.001) and IPI change (*p* = 0.045) in patients who developed PSH compared with those who did not (Table 3).

**Figure 1:**
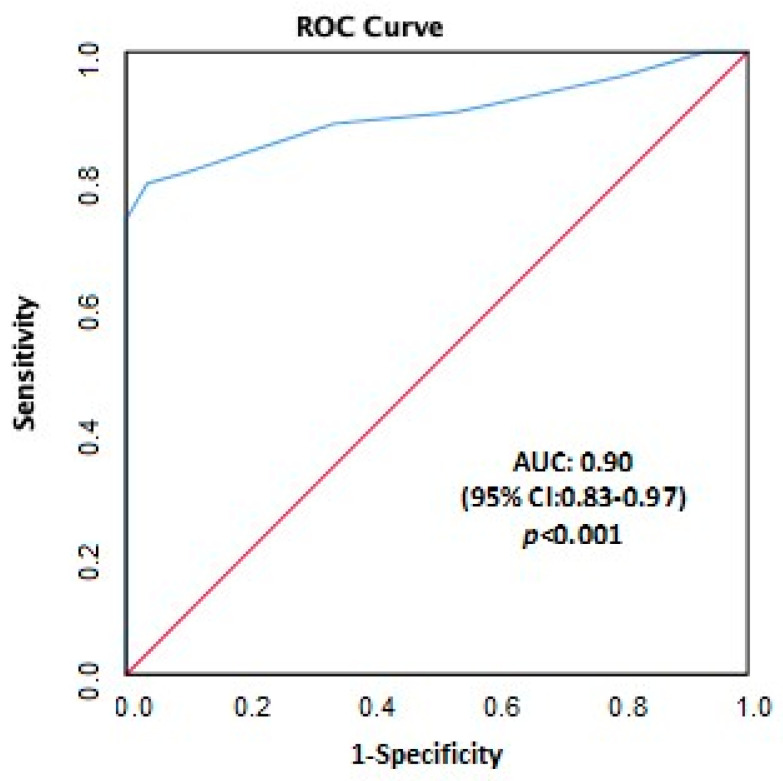
Diagnostic decision-making feature of the amount of change in EtCO_2_ in predicting PSH. Blue line: Line of the amount of change in EtCO_2_; Pink line: Reference line. (ROC curve analysis; AUC: area under the curve; CI: confidence interval).

The independent effects of the significant variables on the prediction of PSH between those who developed PSH and those who did not were evaluated using logistic regression analysis. It was found that the EtCO_2_ difference had an independent effect on the prediction of PSH (*p* < 0.05), whereas age and IPI difference did not (*p* > 0.05) (Table 4). One unit decrease in EtCO_2_ from the baseline increased the risk of PSH by 3.3 times. ROC curve analysis showed that the magnitude of change in EtCO_2_ was diagnostic for predicting PSH (AUC: 0.90 (0.83–0.97; *p* < 0.001)) (Figure 1). The cut-off value for the amount of change in EtCO_2_ was established at 2.5, and the sensitivity, specificity, and positive and negative predictive values were computed in accordance with this value. Using the cut-off value of 2.5 for the EtCO_2_ difference, the sensitivity was 80.8%, the specificity was 90.0%, the positive predictive value was 93.3% and the negative predictive value was 73.0%.

## 4. Discussion

The results of our study showed that the IPI difference did not predict PSH, but the amount of change in EtCO_2_ was diagnostic in predicting PSH. The cut-off value for EtCO_2_ was 2.5. According to this cut-off value, the sensitivity of EtCO_2_ in predicting PSH was 80.8%, the specificity was 90%, the positive predictive value was 93.3% and the negative predictive value was 73%.

The incidence of PSH in our study was 63.4%. The incidence of PSH ranged from 7.4% to 74.1% according to the results of 63 studies reviewed by Klohr et al. Such a wide range of incidence was attributed to the use of 15 different definitions of hypotension in the studies [1]. The most widely used definition of PSH in the literature is a drop in systolic blood pressure of at least 20% from the baseline or a drop below 100 mmHg [11], and this definition of hypotension was employed in our investigation.

Spinal anaesthesia is the standard of care for elective caesarean section, but spinal anaesthesia-induced hypotension remains a major concern. The accurate prediction of hypotension can alter clinical decision making and facilitate early intervention [12]. Therefore, studies have been conducted to investigate possible predictors of PSH and their predictive value during a caesarean section [12,13,14,15,16]. To our knowledge, this is the first study to investigate the effect of EtCO_2_ and IPI in predicting postspinal hypotension.

EtCO_2_ evaluates the effectiveness of ventilation, but the transfer of CO_2_ to the pulmonary system is reliant on cardiac output. Therefore, studies evaluating EtCO_2_ and cardiac output have been performed [17,18,19,20]. Deakin et al. reported that EtCO_2_ was directly related to cardiac output in trauma patients without ventilatory compromise and that mortality was higher in patients with low EtCO_2_. Bulger et al. showed that a prehospital EtCO_2_ of less than 25 mmHg was predictive of haemorrhagic shock in trauma patients [21,22]. In our study, the EtCO_2_ levels measured at the time of PSH development were significantly lower in the hypotensive group compared with the baseline (*p* < 0.001). The difference in the EtCO_2_ change was also greater in those who developed PSH than in those who did not (PSH: 5.5 ± 3.2 vs. non-PSH: 0.7 ± 1.6). In our study, the magnitude of change in EtCO_2_ was found to be a diagnostic decision maker in predicting PSH. In regression analysis, the sensitivity was 80.8%, specificity 90%, positive predictive value 93.3% and negative predictive value 73% according to the cut-off value of 2.5 for the EtCO_2_ difference. In a study of EtCO_2_ monitoring during exercise, the sensitivity of EtCO_2_ in predicting inadequate cardiac output was 76.6% and the specificity was 75% [23]. In studies evaluating the haemodynamic response to fluid replacement, the EtCO_2_ cut-off (2 mmHg) is similar to that of our study, and an increase in EtCO_2_ of 2 mmHg or more after fluid replacement is indicative of intravascular volume deficit [24,25].

IPI has only been used to assess respiratory events and has been shown to be predictive in some cases. Kaur et al. performed IPI monitoring for 48 h in patients extubated in the intensive care unit and reported that IPI predicted failed extubation and a negative change in IPI 1 h after extubation increased the likelihood of failure [25]. It has been reported that IPI = 1 in sedated patients for PEG placement showed a good sensitivity of 81% for hypoxic events, but its specificity was low and the IPI was not superior to isolated EtCO_2_ monitoring [4,5]. In the study reporting that IPI has a high predictive value for pulmonary embolism in patients presenting to the emergency department with dyspnoea, the IPI cut-off value was ≤2, and the sensitivity and specificity of IPI for this value were 100.0% and 96.0%, respectively [26]. In contrast with the respiratory studies in the literature, one study compared the IPI with other cardiac risk scores in acute coronary syndrome and found that the IPI score had a sensitivity of 83.0% and a specificity of 74.3% in predicting major cardiovascular events [27]. In our study, IPI showed no predictive value, but there was a significant difference between patients who developed hypotension and those who did not. Therefore, the interpretation of IPI together with EtCO_2_ may be more meaningful for clinical assessment than the evaluation of IPI alone. The IPI was designed to assess respiratory events, and in fact, when scoring, the relationship between EtCO_2_ and RR was first determined and scored, and then the effect of SpO_2_ on this score was calculated. The HR was constructed as a secondary factor in all these evaluations [3]. In patients undergoing a caesarean section under spinal anaesthesia, the HR is often unstable; tachycardia as a reflex response to hypotension or bradycardia due to high levels of spinal anaesthesia may be seen. The fact that the HR is regulated as a secondary factor in the IPI algorithm may be an obstacle to predicting an unstable haemodynamic state. A new algorithm that prioritises HR and EtCO_2_, which is known to reflect cardiac output well, can be used as an early warning system for haemodynamic instability.

In our study, although the mean age in the group that developed PSH was younger than the other group, logistic regression analysis showed that age had no predictive value for PSH. Similarly to our findings, most studies in the literature have reported that maternal age, weight, height and BMI have no predictive value for PSH [14,16,28,29], while fewer studies have suggested a weak correlation between BMI and PSH among demographic data [2,30].

In both patients with and without PSH, an increase in SpO_2_ was observed with a decrease in blood pressure. Similarly, Crooks et al. demonstrated an inverse relationship between blood pressure and pulse oximetry accuracy. It has been reported that at low blood pressure, the signal integrity received from translucent tissue is compromised due to poor peripheral perfusion, resulting in the false overestimation of pulse oximetry readings [31].

In our study, the time from spinal anaesthesia induction to the onset of PSH was approximately 3 min. In the study by Keera et al., this time was 5.6 min [32]. A study of 227 pregnant women reported that hypotension developed in the first 5 min in most patients (56%) [33].

The mean time from the onset of PSH to a normotensive state was 3.2 ± 1.4 min in our study. In a study in which ephedrine was administered as a bolus, the duration of the hypotension was 6.5 ± 4.26 min [32], and in another study in which ephedrine was administered both as an infusion and bolus, the duration of the hypotension was 2.6 ± 0.5 min [34]. The duration of hypotension is more important than its depth. Hypotension lasting less than 2 min has been reported to cause no decrease in the neonatal Apgar score or neurobehavioural effects, whereas hypotension lasting more than 4 min has been reported to cause a decrease in the Apgar score and neurobehavioural changes [11].

## 5. Limitations

The measured RR and HR may have been influenced by pre- and intra-operative anxiety, or ephedrine. In addition, there may have been inaccurate measurements of the RR due to intraoperative abdominal manoeuvres. All of these factors may have influenced the EtCO_2_ and IPI values.

Two methods, mainstream and sidestream, are used to measure EtCO_2_. The microstream capnography used in our study is actually a sidestream system. The working principle of the sidestream system is based on the collection of exhaled air samples using a sampling tube. The handicap in this system is that the EtCO_2_ is likely to be measured lower because the sampling gas can be diluted with air. Further studies comparing mainstream and microstream capnography in predicting postspinal hypotension can be performed.

In our study, blood pressure was measured every 2 min, while the EtCO_2_ and IPI were measured every 5 s. It would have been healthier in terms of assessing the correlation if the blood pressure had also been measured immediately. This would have required invasive arterial blood pressure monitoring, which we did not consider to be appropriate because it is invasive. With arterial blood pressure monitoring, the relationship between EtCO_2_ and hypotension could have been observed instantaneously. We did not choose arterial blood pressure monitoring because it is an invasive procedure and is not part of the routine in obstetric anaesthesia.

In our study, we concluded that hypotension can be detected early with the use of capnometry, but it is not known to what extent the use of monitors reduces the side effects associated with PSH. Therefore, in future studies, we need to create groups with and without the use of monitors, create a hypotension treatment plan according to EtCO_2_ and IPI values, and evaluate the effect of all this on maternal/fetal outcomes.

## 6. Conclusions

The IPI showed no predictive value for postspinal hypotension in cesarean sections. However, EtCO_2_ monitoring, which is non-invasive and real-time monitoring, can be used to predict postspinal hypotension so that PSH can be recognised immediately without waiting for the non-invasive blood pressure measurement time.

Non-invasive and real-time monitoring of EtCO_2_ and IPI can be used to predict postspinal hypotension in caesarean sections. Thus, PSH can be recognised immediately, and early intervention is possible.

## Figures and Tables

**Table 1:** Comparison of demographic data and baseline IPI and its components of patients with and without PSH.

	Postspinal Hypotension		*p*
	Developed (*n* = 52)	Non-Developed (*n* = 30)	
**Age (year)**	28.4 ± 5.6 (17–42)	31.3 ± 5.6 (20–41)	0.025 *^a^
**Weight (kg)**	79.9 ± 12.9 (56–109)	80.8 ± 14.1 (60–110)	0.772 ^a^
**Height (cm)**	160.6 ± 6.6 (150–175)	162.6 ± 6.0 (150–173)	0.175 ^a^
**BMI (kg/m^2^)**	30.9 ± 4.5 (21.5–42.2)	30.6 ± 5.2 (20.8–39.1)	0.743 ^a^
**Obesity Class**			
Normal weight	3 (5.8)	3 (10.0)	
Overweight	18 (34.6)	12 (40.0)	0.628 ^b^
Obese	31 (59.6)	15 (50.0)	
**Co-morbidity**	6 (11.5)	6 (20.0)	0.341 ^c^
**Gestational HT**	1 (1.9)	1 (3.3)	1.000 ^c^
**Gestational DM**	2 (3.8)	1 (3.3)	1.000 ^c^
**EtCO_2_ (mmHg)**	26.8 ± 2.7 (14–32)	26.5 ± 2.0 (23–30)	0.835 ^b^
**RR/min**	22.6 ± 5.8 (10–36)	23.0 ± 5.6 (14–38)	0.738 ^a^
**SpO_2_ (%)**	98.1 ± 1.0 (96–100)	97.4 ± 1.9 (91–100)	0.044 *^b^
**HR/min**	97.9 ± 17.5 (58–139)	91.7 ± 13.0 (66–128)	0.097 ^a^
**IPI**	7.4 ± 1.4 (2–10)	7.5 ± 1.2 (5–10)	0.773 ^a^

PSH: postspinal hypotension. Continuous variables are presented as “mean ± standard deviation (minimum–maximum)” and categorical variables are presented as “number (percentage)”. BMI: body mass index; HT: hypertension; DM: diabetes mellitus; EtCO_2_: end-tidal carbon dioxide; RR: respiratory rate; SpO_2_: peripheral oxygen saturation; HR; heart rate; IPI: integrated pulmonary index. ^a^ Student’s *t*-test; ^b^ Pearson chi-square test; ^c^ Fisher’s exact test; * *p* < 0.05.

**Table 2:** Comparison of baseline values of IPI and its components with values during the development of PSH in women with PSH.

PSH (*n* = 52)	Basale	PSH Development Time	*p*
	Mean ± SD (Min–Max)	Mean ± SD (Min–Max)	
**EtCO_2_ (mmHg)**	26.8 ± 2.7 (14–32)	21.3 ± 3.6 (12–31)	<0.001 *^b^
**RR/min**	22.6 ± 5.8 (10–36)	21.0 ± 5.7 (11–36)	0.104 ^a^
**SpO_2_ (%)**	98.1 ± 1.0 (96–100)	98.8 ± 1.3 (94–100)	<0.001 *^b^
**HR (beats/min)**	97.9 ± 17.5 (58–139)	95.8 ± 28.6 (51–176)	0.590 ^a^
**IPI**	7.4 ± 1.4 (2–10)	6.5 ± 1.9 (2–9)	0.002 *^b^

PSH: postspinal hypotension; SD: standard deviation; EtCO_2_: end-tidal carbon dioxide; RR: respiratory rate; SpO_2_: peripheral oxygen saturation; HR: heart rate; IPI: integrated pulmonary index. ^a^ Paired sample *t*-test; ^b^ Wilcoxon signed rank test; * *p* < 0.05.

**Table 3:** Change in hemodynamic parameters and IPI and its components measured during the development of PSH compared with baseline values.

	Postspinal Hypotension		*p* ^a^
	Developed (*n* = 52)	Non-Developed (*n* = 52)	
	Mean ± SD (Min–Max)	Mean ± SD (Min–Max)	
**SBP (mmHg)**	41.6 ± 17.2 (13–90)	11.4 ± 8.5 (−8–32)	<0.001 *
**DBP (mmHg)**	34.0 ± 12.8 (1–65)	10.9 ± 8.2 (−5–26)	<0.001 *
**MAP (mmHg)**	37.3 ± 13.8 (3–72)	10.4 ± 8.0 (−5–29)	<0.001 *
**EtCO_2_ (mmHg)**	5.5 ± 3.2 (−1–15)	0.7 ± 1.6 (−3–4)	<0.001 *
**RR (/dk)**	1.6 ± 7.0 (−18–18)	−0.9 ± 1.6 (−8–1)	0.097
**SpO_2_ (%)**	−0.7 ± 1.4 (−3–5)	−0.8 ± 1.6 (−8–1)	0.441
**HR (beats/min)**	2.0 ± 27.1 (−71–56)	−3.8 ± 13.4 (−45–19)	0.164
**IPI**	0.9 ± 1.9 (−3–7)	0.1 ± 1.1 (−2–2)	0.045 *

PSH: postspinal hypotension; SBP: systolic blood pressure; DBP: diastolic blood pressure; MAP: mean arterial pressure; EtCO_2_: end-tidal carbon dioxide; RR: respiratory rate; SpO_2_: peripheral oxygen saturation; HR; heart rate; IPI: integrated pulmonary index. ^a^ Mann--Whitney U test; * *p* < 0.05.

**Table 4:** Independent effect of age, ETCO_2_ and IPI in predicting PSH.

	OR (%95 CI)	*p*
**Age (year)**	0.70 (0.47–1.05)	0.083
**EtCO_2_ (mmHg)**	3.30 (1.24–8.81)	0.017 *
**IPI**	0.34 (0.09–1.25)	0.624

PSH: postspinal hypotension; EtCO_2_: end-tidal carbon dioxide; IPI: integrated pulmonary index; OR: odds ratio; CI: confidence interval; * *p* < 0.05.

## Data Availability

The data presented in this study are available on request from the corresponding author.
